# Micro/Nanoplastics and Periodontitis: An Environmental Microbiology Perspective on Oral Retention and Systemic Risk

**DOI:** 10.3390/microorganisms14051014

**Published:** 2026-04-30

**Authors:** Mark Cannon, John Peldyak, Paul Reynolds

**Affiliations:** 1 Ann and Robert H Lurie Children’s Hospital, Northwestern University, Chicago, IL 60611, USA; 2 School of Dentistry, University of Michigan, Ann Arbor, MI 48109, USA; 3 Department of Cell Biology and Physiology, Brigham Young University, Provo, UT 84602, USA

**Keywords:** microplastics, nanoplastics, periodontitis, oral biofilm, dental calculus, chronic low-grade inflammation, plastisphere, environmental microbiology, oral-systemic health

## Abstract

Micro- and nanoplastics (MNPs) have now been detected in human blood, placenta, and arterial tissue, yet the oral cavity has received strikingly little mechanistic attention despite serving as a primary portal of environmental exposure and a local site of polymer generation from dental and oral-care materials. This narrative review addresses that gap from an environmental microbiology perspective, synthesizing recent literature on periodontal disease, chronic low-grade inflammation, oral biofilms, dental materials, microbial–plastic interactions, and systemic chronic disease risk. Unlike prior reviews, we apply an explicit three-tier evidentiary framework (established, plausible, unproven) that distinguishes what is directly demonstrated from what is biologically plausible but unproven, and we situate the periodontal environment specifically as a particle-retention and inflammatory-amplification niche. The strongest direct oral evidence shows that human dental calculus harbors at least 26 microplastic types, dominated by polyamide (41.4%), polyethylene (32.7%), and polyurethane (7.0%). Polyethylene isolated from calculus induces cytotoxicity, apoptosis, impaired migration, NF-κB activation, and upregulation of IL-1β and IL-6 in human gingival fibroblasts. From a microbiological standpoint, oral organisms actively degrade methacrylate dental polymers, and the degradation products of these polymers reciprocally modulate oral bacterial virulence gene expression. Across experimental systems, MNPs activate oxidative stress, inflammasome signaling, macrophage polarization, and barrier dysfunction, pathways that overlap extensively with periodontal pathobiology. Adjacent environmental microbiology demonstrates that plastic-associated biofilms enhance extracellular polymeric substance production, quorum sensing, pathogen persistence, and antibiotic resistance gene transfer, supporting a plausible but not yet validated oral plastisphere within plaque and calculus. We argue that periodontitis should be reconceptualized as a chronically inflamed particle-processing interface that may increase local MNP retention, cellular reactivity, and systemic inflammatory spillover, with implications for cardiovascular, metabolic, and other chronic disease risk pathways. Current evidence does not yet prove that environmental MNP exposure causes human periodontitis, and that evidentiary boundary is maintained throughout. A priority research agenda is proposed, centered on contamination-controlled subgingival biomonitoring stratified by periodontal status, spatially resolved multi-species biofilm models, polymer source attribution, and longitudinal clinical studies linking oral plastic burden to inflammatory and systemic outcomes.

## 1. Introduction

Micro- and nanoplastic (MNP) pollution is now understood not only as a marine or terrestrial contamination problem, but also as a biologically relevant exposure issue at human epithelial interfaces. Most discussions have focused on ingestion, inhalation, intestinal toxicity, pulmonary injury, and translocation into blood or distal organs. The oral cavity has received comparatively little mechanistic attention, even though it is the first compartment to contact food, beverages, airborne particles, oral-care products, and many polymer-containing medical and consumer materials [1,2,3].

In quantitative terms, the gastrointestinal tract is generally considered the dominant route of human MNP intake, with dietary estimates on the order of 10^4^–10^5^ particles per person per year, followed by inhalation, which delivers airborne fibers and fragments to the upper and lower airways with measurable pulmonary deposition [4,5,6]. The oral cavity is frequently conflated with the GI tract, yet it is mechanistically distinct. It is the first mucosal interface contacted by ingested and inhaled particles; it uniquely combines environmental exposure with locally generated polymer debris from dental and oral-care materials, and it hosts a structured, chronically inflamed biofilm environment in periodontitis that is not replicated elsewhere in the alimentary tract. These features justify treating the oral cavity as a distinct exposure compartment rather than a passive conduit to the gut.

Periodontitis is a chronic dysbiotic inflammatory disease of the tooth-supporting tissues. It is driven by a structured oral biofilm and by a maladaptive host response that promotes collagen breakdown, pocket formation, attachment loss, and alveolar bone destruction. Beyond local tissue damage, periodontitis is increasingly framed as a source of chronic low-grade systemic inflammation, with cytokine spillover and plausible contributions to cardiovascular, metabolic, and other chronic conditions [7,8,9]. The reference to “silent inflammation” reflects common lay usage; throughout this review, we use the scientifically preferred term chronic low-grade inflammation to denote subclinical inflammatory signaling that persists beyond overt acute symptoms.

The overlap between periodontitis and MNP biology is compelling for four reasons, the first two now supported by direct oral evidence. First, the oral cavity is both an exposure portal and a local source of plastic particles: chewing gum releases measurable MNPs into saliva [3], oral-care products contribute polymer debris [10], and dental and orthodontic materials degrade, abrade, or leach polymeric by-products [11,12,13,14]. Second, retention of plastic particles within the oral cavity has now been directly demonstrated: human dental calculus harbors at least 26 microplastic types, dominated by polyamide (41.4%), polyethylene (32.7%), and polyurethane (7.0%) [15], confirming that the periodontal environment—rich in biofilm biomass, mineralized plaque, proteases, oxidative stress, and altered epithelial integrity—acts as a particle-retention niche rather than a passive conduit. Third, MNP toxicology and periodontal pathobiology share overlapping mechanisms in directly relevant cells: polyethylene particles isolated from calculus activate NF-κB and upregulate IL-1β and IL-6 in human gingival fibroblasts [15], consistent with broader evidence for ROS, inflammasome signaling, macrophage polarization, barrier dysfunction, and impaired repair across MNP toxicology [2,16]. Fourth, environmental microbiology has shown that plastic-associated biofilms can enrich pathogenic taxa, virulence traits, extracellular polymeric substance (EPS) production, and antibiotic resistance gene (ARG) exchange, raising the possibility that plastic particles embedded in oral biofilms could intensify dysbiosis [17,18,19,20].

This review was therefore designed to rephrase the question from an environmental microbiology perspective: Can periodontitis be understood as a chronically inflamed oral microenvironment in which microplastics and nanoplastics are retained, transformed, or biologically amplified? We analyzed the current PubMed-indexed evidence on oral exposure routes, direct periodontal findings, chronic inflammatory mechanisms, plastisphere biology, polymer biodegradation by oral microbes, and the implications for chronic disease. Throughout, we distinguish what is directly demonstrated from what is mechanistically plausible yet to be studied.

Despite growing interest in MNPs at human epithelial interfaces, the existing literature has several clear shortcomings specific to the oral compartment. First, oral MNP exposure is frequently collapsed into the gastrointestinal route, obscuring the distinct retention, microbial, and inflammatory dynamics of the oral cavity. Second, most mechanistic evidence derives from gut, pulmonary, or generic human cell systems, with very few studies in periodontal or gingival cell models. Third, polymer source attribution in the oral cavity (environmental versus dentistry-derived) is largely unaddressed, despite its importance for causal interpretation. Fourth, the environmental microbiology literature on plastic-associated biofilms has not been systematically applied to oral biofilms, leaving the oral plastisphere concept untested. Fifth, longitudinal human data linking oral MNP burden to periodontal progression or systemic inflammatory outcomes are absent. This review was designed to address these gaps by integrating oral, mechanistic, microbiological, and systemic evidence within an explicit evidentiary framework, and by proposing a prioritized research agenda to move the field from plausibility to empirical test.

## 2. Literature Search Strategy and Scope

A structured narrative review approach was used. PubMed was searched through 7 March 2026, using combinations of the following terms: “microplastics,” “nanoplastics,” “micro- and nanoplastics,” “oral cavity,” “mouth,” “saliva,” “dental calculus,” “plaque,” “oral biofilm,” “periodontitis,” “periodontal disease,” “gingival fibroblasts,” “chronic inflammation,” “plastisphere,” “biofilm formation,” “antibiotic resistance gene transfer,” “dental resin,” “dental composite,” “oral bacteria,” and “polymer degradation.” Additional PubMed searches targeted chronic disease contexts (blood, placenta, arteries, gut, lung) when direct periodontal evidence was limited.

Priority was given to PubMed-indexed primary research in humans, mammalian models, oral cell systems, and mechanistic biofilm studies. Recent reviews were included when they were useful for framing evidence quality, definitions, and research gaps. Because direct oral and periodontal MNP studies remain limited, adjacent evidence from environmental microbiology, intestinal toxicology, pulmonary toxicology, and dental materials science was incorporated only when it clarified a biologically plausible pathway that could reasonably extend to the periodontium. PubMed was selected as the sole database because the review is explicitly biomedical and mechanistic in scope, PubMed indexing provides the most consistent coverage of primary human, clinical, and molecular studies relevant to periodontology and MNP toxicology, and single-database sourcing supports search reproducibility for a narrative synthesis. We acknowledge that this approach may underrepresent environmental engineering and materials science studies, which are indexed preferentially in Scopus or Web of Science; a fully systematic review across multiple databases is identified in Section 11 as a necessary next step.

Studies were included when they met the following criteria: (i) peer-reviewed primary research, authoritative review, or indexed editorial synthesis retrievable through PubMed; (ii) published in English; (iii) direct relevance to at least one of the review’s thematic domains—oral/periodontal MNP exposure or retention, mechanistic MNP toxicology in human or mammalian systems, oral microbial–polymer interactions, plastisphere biology with translational implications, or oral–systemic inflammatory pathways; and (iv) methodological transparency sufficient to evaluate the strength of the evidence claim being extracted. Studies were excluded when they were non-peer-reviewed (preprints, conference abstracts without full methods, opinion pieces without sourcing), limited to purely environmental distribution or remediation data with no biological or health translation, or duplicative of stronger primary evidence already represented in the review. Where multiple studies addressed the same mechanistic endpoint, preference was given to those in oral or periodontal systems, followed by human cell systems, followed by mammalian in vivo models, followed by environmental biofilm systems. This hierarchical prioritization is reflected in the tiered evidence classification defined above and summarized in Table 1.

To support transparency, each claim discussed in this review was assigned to one of three evidentiary tiers. *Established* claims are those supported by direct human or human-cell evidence in an oral or periodontal context, with independent replication or mechanistic confirmation (e.g., detection of microplastics in human dental calculus; cytokine induction in human gingival fibroblasts). *Plausible* claims are those supported by consistent mechanistic evidence in adjacent biological systems (environmental plastisphere biology, intestinal or pulmonary MNP toxicology, non-oral human cell models) for which direct oral or periodontal validation is lacking. *Unproven* claims are those requiring longitudinal or interventional human data that do not yet exist, most notably any causal claim that environmental MNP exposure produces human periodontitis. This tiering is applied throughout the narrative and is summarized in the “Strength of support” column of Table 1.

This is not a systematic review or meta-analysis, and no attempt was made to pool effect sizes. The aim was instead to produce an accurate translational synthesis that is aware of evidentiary boundaries. In practical terms, the present literature supports a mechanistic model linking MNPs to periodontal inflammation, but not a definitive causal claim that environmental MNP exposure causes human periodontitis.

## 3. Periodontitis as a Chronic Low-Grade Inflammatory Interface

The microbial ecology of periodontitis is not simply an increase in total bacterial load; it involves a shift toward a dysbiotic biofilm with altered metabolic activity, virulence expression, and host interactions. Metagenomic and metatranscriptomic work shows that periodontitis is associated not only with compositional differences in plaque communities but also with altered species-specific gene expression across oral sites [21]. Saliva can reflect changes in subgingival communities and track disease severity and treatment response, reinforcing the view that periodontitis extends beyond a single niche into a broader oral ecological state [22]. The conceptual basis for this dysbiotic shift is now well established: keystone pathogens such as *Porphyromonas gingivalis*, despite being low in total abundance, can subvert complement-mediated immune surveillance to remodel the entire community quantitatively and qualitatively, enabling pathobionts to exploit inflammatory tissue breakdown products as nutrients and sustain destruction disproportionate to their biomass [23]. This polymicrobial synergy model—in which inflammation and dysbiosis reinforce each other—is the mechanistic foundation for the particle-retention and inflammatory-amplification model proposed in this review.

Clinically, the periodontal pocket is a chronically inflamed interface where bacterial biomass, host proteases, neutrophil activity, cytokines, bleeding, and tissue breakdown coexist. Reviews of oral-systemic health consistently describe periodontitis as a contributor to a low-grade systemic inflammatory burden, while longitudinal human data support links between periodontal disease and systemic outcomes, such as hypertension, through inflammatory pathways [7,9]. The cytokine milieu is dominated by mediators central to MNP toxicology, including IL-1β, IL-6, TNF-α, and inflammasome-associated signals [8].

This inflammatory background is relevant to particle biology. The oral cavity is not a passive conduit. It contains pellicle-coated surfaces, salivary proteins, mucinous macromolecules, dynamic shear forces, structured biofilms, and, in disease, an inflamed and more permeable epithelial barrier. These features can influence adhesion, agglomeration, residence time, and cellular access of exogenous particles. In other words, periodontitis may modify the local tissue dose of retained particles even when ambient environmental exposure is unchanged.

For the present review, periodontitis is therefore conceptualized as a chronic inflammatory particle-processing niche. This framing does not imply that plastics are necessary for disease, nor that all periodontal inflammation requires environmental pollutants. Instead, it recognizes that an inflamed periodontal ecosystem may be uniquely capable of retaining, reacting to, and perhaps disseminating MNPs or plastic-derived by-products.

While limited, direct evidence supporting this framing is convergent across three independent lines. First, human dental calculus has been shown to retain at least 26 microplastic types, establishing that the periodontal environment physically accumulates polymer particles rather than merely transiting them [15]. Second, polyethylene particles recovered from the calculus context are biologically active in the exact cell type responsible for periodontal tissue maintenance: they reduce gingival fibroblast viability, impair migration, and activate NF-κB, leading to upregulation of IL-1β and IL-6 [15]. Third, calculus itself is not inert; it stimulates IL-1β secretion through NLRP3 inflammasome activation in human and mouse phagocytes [24], meaning a calculus matrix containing polymer particles represents a composite inflammatory substrate rather than a passive reservoir. Taken together, these data support the biological plausibility of the particle-processing niche as a working model, while leaving its clinical implications, particularly whether higher oral MNP burdens accelerate periodontal breakdown, to be tested in the longitudinal designs outlined in Section 11.

## 4. The Oral Cavity as an Exposure Portal and Local Source of Micro-/Nanoplastics

Direct oral exposure to plastic particles can arise from everyday consumer products and from dentistry itself. A recent analytical study demonstrated the release of MNPs into the oral cavity from chewing gum, highlighting that common products can contribute directly measurable oral exposure [10]. A broader review of oral-care products similarly argued that toothpastes, mouthwashes, brushes, and other routine products may serve as underrecognized sources of orally delivered MNPs [11].

Specific oral hygiene products deserve explicit mention because they are used daily and their contributions are underrecognized relative to dentistry-based exposure. Toothbrush bristles, predominantly polypropylene and nylon, shed microplastic fragments during normal brushing, with estimated intraoral exposure exceeding 2 × 10^6^ particles per person per year [11]. Dental floss is typically manufactured from nylon or polyester, and many widely used brands are coated with polytetrafluoroethylene (PTFE), a polyfluoroalkyl substance that can shed both MPs and associated PFAS during interproximal use. Mouthwash formulations may contain suspended polymer particles and are delivered directly to oral mucosal and subgingival surfaces, with residence patterns distinct from solid products. These exposures occur at sites most relevant to periodontal health: the gingival margin, interproximal surfaces, and subgingival sulcus. Their cumulative contribution to oral MNP burden in periodontal disease has not been quantified.

Dentistry adds a second, conceptually distinct pathway. The oral cavity contains polymeric restorations, adhesives, sealants, prosthetic components, aligners, retainers, and orthodontic appliances, all of which are subject to abrasion, thermal cycling, enzymatic attack, pH fluctuations, and bacterial colonization. Dentistry has been framed as both an exposure source and a translational setting in which oral and environmental plastic burdens intersect [1]. Orthodontic materials can generate microplastics and nanoplastics with measurable immunologic effects on macrophage differentiation and homeostasis [14].

This dual-source problem is central to the interpretation of oral findings. When plastics are detected in the mouth, they may derive from food packaging, indoor dust, drinking water, airborne deposition, oral-care products, or dental materials themselves. The mouth is therefore not only an exposure gateway but also a microreactor where environmental particles and locally generated polymer debris can mix. From a periodontal perspective, that mixture is clinically relevant because even non-environmental polymer fragments may still amplify local inflammation and biofilm pathogenicity.

A key implication is that future oral biomonitoring studies must distinguish external environmental particles from dentistry-derived polymers whenever possible. That distinction is not a minor methodological detail; it is essential for causal interpretation. A mouth with extensive resin-based restorations or orthodontic therapy may exhibit a fundamentally different polymer signature from a mouth without such materials, even if ambient environmental exposure is similar.

A rough quantitative comparison helps place these sources in context, though cross-study comparison is limited by heterogeneous detection platforms, size cutoffs, and reporting units. Chewing gum has been estimated to release hundreds to thousands of MPs per piece into saliva over a one-hour chewing interval, with polymer identification confirming synthetic gum bases as the dominant source [10]. Oral-care products are estimated to contribute on the order of 10^5^ to 10^6^ particles per person per year from toothpaste and toothbrushes alone, with polyethylene, ethylene-vinyl acetate, and polypropylene as predominant polymers [11]. Orthodontic appliances and clear aligners release measurable MPs during mechanical cycling, with immunologically active effects on macrophage differentiation reported for orthodontic-derived particles [14]. Resin-based restorative materials contribute an episodic burden linked to placement, finishing, and daily wear, although polymer-specific release rates in vivo remain poorly quantified [12,13]. On current evidence, consumer product exposure (gum and oral care) appears to dominate by particle count, while dentistry-derived exposure is more variable between individuals and more likely to produce polymer signatures distinct from the environmental background. Distinguishing these contributions at the individual level is a core requirement of the biomonitoring agenda in Section 11.

## 5. Direct Evidence for Oral Retention: Saliva, Plaque, and Dental Calculus

At present, the most important direct human evidence comes from dental calculus. Human dental calculus has been reported to contain at least 26 microplastic types, with polyamide (41.4%), polyethylene (32.7%), and polyurethane (7.0%) as predominant components [15]. This is a major finding because it shows that at least some MNPs are not merely transient oral visitors; they can be retained within a calcified plaque matrix that persists over time.

Methodologically, Wu et al. identified polymers using laser-direct infrared (LDIR) and micro-FTIR spectroscopy with commercial polymer library matching on a particle-by-particle basis [15]. Contamination controls included procedural blanks, pre-filtered reagents, avoidance of plastic labware, and laminar-flow sample handling, with polymer assignments retained only when spectral match quality exceeded the platform’s confidence threshold. These safeguards are standard for current MNP biomonitoring but are less sensitive for sub-10 µm and nanoscale particles, where Raman microspectroscopy with enhanced substrates (e.g., SERS) offers higher resolution, reinforcing the multi-platform biomonitoring agenda outlined in Section 11. The same study went beyond detection. Polyethylene MNPs reduced the viability of human gingival fibroblasts, increased apoptosis, impaired migration, activated NF-κB signaling, and increased the expression of IL-1β and IL-6 [15]. From a periodontal viewpoint, these effects are highly relevant: gingival fibroblasts contribute to extracellular matrix maintenance, wound healing, and inflammatory signaling. A particle that both drives cytokine production and impairs fibroblast migration could, at least in principle, delay repair of inflamed periodontal tissues and increase their propensity for chronicity.

The translational weight of these in vitro findings depends on whether the exposure concentrations tested approximate those encountered by gingival fibroblasts in vivo. The study reported dose-dependent cytotoxicity, impaired migration, and inflammatory activation, with effect magnitudes scaling with exposure concentration [15]. As is typical across the current MNP in vitro literature, however, the concentrations used in cell culture are generally higher than would be expected from ambient oral exposure alone, reflecting the practical need to produce measurable endpoints within short experimental windows. Physiologically relevant tissue-level concentrations in inflamed periodontal tissue have not been directly quantified, and any extrapolation from saliva, plaque, or calculus burden to gingival interstitial dose remains uncertain. This limitation is not unique to this study; it applies broadly to MNP toxicology and reinforces the priority assigned in Section 11 to contamination-controlled in vivo periodontal biomonitoring, which is the only route to resolving whether observed in vitro effects occur at concentrations actually achieved in diseased periodontal tissue.

Dental calculus itself is already known to be biologically active rather than inert. Dental calculus can stimulate IL-1β secretion through NLRP3 inflammasome activation in human and mouse phagocytes [24]. This means that a calculus matrix containing bacteria, mineral crystals, endotoxin, and now demonstrable plastic particles may represent a composite inflammatory substrate. The calculus–plastic combination is thus more important than either component considered alone.

The long-term reservoir concept is also supported by work outside the plastic field. Dental calculus preserves a rich metabolomic record in modern and historic samples, reinforcing the idea that calculus acts as a stable archive of oral exposures and biomolecules [25]. By analogy, calculus may serve as a cumulative exposure matrix for polymer particles and associated chemicals, although the kinetics of trapping, mineralization, and release remain largely unknown.

Whether calculus retention reflects active biological sequestration or passive physicochemical accumulation is an open and interpretively important question. A passive model would hold that MNPs in saliva, food, and dentifrice are incidentally entrapped during mineralization of plaque, with retention largely a function of particle size, surface charge, and the kinetics of calcium phosphate deposition. An active model would hold that biofilm constituents, including extracellular polymeric substances, salivary pellicle proteins, and bacterial surface structures, preferentially adsorb, concentrate, or coat polymer particles before or during mineralization, producing a polymer signature that reflects biological selection rather than ambient exposure alone. Current evidence cannot fully distinguish these mechanisms, but two observations favor a mixed model: the polymer distribution in calculus (dominated by polyamide, polyethylene, and polyurethane) only partially mirrors the polymer distribution reported in saliva or gum-derived oral exposure [10,15], and calculus-derived polyethylene retains biological activity in gingival fibroblasts [15], suggesting the matrix does not fully neutralize particle reactivity. The distinction matters because a passive reservoir would primarily function as a cumulative exposure archive, whereas active retention would imply that oral biofilm chemistry shapes which polymers persist and remain biologically available, an interpretation with direct consequences for biomonitoring design and for whether mechanical debridement meaningfully reduces inflammatory burden.

Direct evidence is still missing for several clinically important compartments. PubMed-indexed literature remains sparse regarding polymer-specific quantification of MNPs in saliva, supragingival plaque, subgingival plaque, gingival crevicular fluid, or periodontal pocket fluid stratified by periodontal status. The absence of these studies is one of the field’s clearest gaps. Accordingly, current conclusions about “oral biofilm storing plastic” should be considered well-supported for dental calculus, plausible for mature plaque biofilms, and largely untested in the subgingival niche.

## 6. Mechanistic Convergence Between Micro-/Nanoplastic Toxicology and Periodontal Pathobiology

The strongest argument linking MNPs to periodontal disease is mechanistic convergence. Across diverse cell and animal systems, MNPs induce oxidative stress, proinflammatory cytokine production, inflammasome signaling, cell death, immune polarization, and barrier injury. These are the same broad pathways that sustain periodontitis once dysbiosis is established [16,26].

The oral cell data are particularly persuasive because they show these mechanisms in periodontal cells. Polyethylene recovered from the context of human dental calculus activated NF-κB and elevated IL-1β and IL-6 after exposure of gingival fibroblasts [15]. This aligns with the broader periodontal literature, which implicates IL-6 and inflammasome-related signaling in tissue destruction, osteoimmunology, and the propagation of oral–systemic inflammation [8,16].

Human cell experiments outside dentistry support the plausibility of inflammation. Commercial and environmental MNP preparations, especially PET-rich samples, can trigger IL-1β and IL-6 responses and induce cell death in human cells [26]. Although these were not periodontal cells, the findings matter because they indicate that authentic environmental MNP mixtures can be inflammatory at human tissue interfaces. The relevance to the mouth is straightforward: a chronically inflamed periodontal environment would not be expected to be less sensitive than other epithelial or stromal systems.

Barrier dysfunction and impaired repair offer a second bridge. Chen et al. showed that chronic oral exposure to nonphagocytosable polystyrene microplastics in mice disrupted redox balance, shifted immune homeostasis, and injured the colonic barrier via bile acid–microbiota interactions [27]. In periodontal tissues, epithelial integrity and connective tissue repair are already compromised by dysbiosis and excessive host inflammation. The impaired migration of gingival fibroblasts after polyethylene exposure [15] suggests that MNPs could worsen a diseased oral interface by slowing the rate of restitution after injury.

A third bridge is immune polarization. Orthodontic-derived microplastics can be phagocytosed and are associated with changes in macrophage differentiation, favoring a more proinflammatory state [15]. In non-oral models, Skaba et al. summarized evidence for immune disruption [28], while Chen et al. reported Th17/Treg imbalance during chronic microplastic exposure [27]. This is notable because periodontitis is also a disease of dysregulated leukocyte recruitment, cytokine networks, and osteoimmune imbalance. A particle that biases macrophage or T-cell responses toward inflammatory persistence would naturally fit within the context of periodontal pathogenesis.

These parallels do not prove synergy, but they do justify a testable hypothesis: MNP exposure and periodontitis may act additively or even multiplicatively because they converge on overlapping molecular circuits. That hypothesis is stronger than mere ecological speculation, yet still short of clinical demonstration.

Several limitations constrain this mechanistic extrapolation. Most supporting data derive from gut, pulmonary, or generic human cell systems whose particle exposure profiles, matrix interactions, and host-cell composition differ from those of the periodontium. In vitro models rarely capture the coexisting bacterial biomass, mineralized matrix, gingival crevicular fluid, and mechanical shear that define the inflamed periodontal niche, and many use pristine commercial particles rather than weathered or biofilm-embedded polymers. Shared pathway activation across tissues, therefore, establishes biological plausibility, but not periodontal specificity, and the magnitude of effect in authentic periodontal tissue remains an open empirical question.

## 7. The Oral Biofilm as a Potential Plastic Reservoir and Pathogenic Amplifier

Environmental microbiology has shown that plastics do not remain biologically neutral once they enter microbial ecosystems. They are rapidly colonized and transformed into plastic-associated biofilms collectively described as the plastisphere. Recent synthesis shows that microplastic biofilms can act as hotspots for ARGs and potential pathogens, and that their ecological behavior differs from surrounding non-plastic substrates [20].

Primary studies deepen this concern. Polystyrene nanoparticles can promote *Pseudomonas aeruginosa* biofilm formation, increase EPS secretion, stimulate quorum sensing, elevate virulence factor production, and enhance antibiotic resistance [17]. Nanoplastics can induce prophage activation and quorum sensing, thereby enhancing biofilm mechanical and chemical resilience [19]. Microplastic biofilms can increase conjugative ARG transfer frequencies in estuarine systems [18]. The plastisphere can protect *Salmonella typhimurium* from ultraviolet stress under simulated environmental conditions [29]. Finally, clinical *Pseudomonas aeruginosa* isolates that encode plastic-degrading enzymes can survive on plastic as the sole carbon source and exhibit enhanced biofilm formation and pathogenicity [30].

These are not oral studies, but their translational significance is substantial. Oral plaque is a densely structured, multispecies biofilm with strong matrix dependence, nutrient gradients, quorum sensing, intense horizontal microbial interactions, and repeated exposure to host antimicrobials. If microplastic or nanoplastic particles become embedded within plaque or mineralize into calculus, they may create microhabitats that resemble miniature plastisphere niches. Such niches could alter local oxygen gradients, host protein adsorption, matrix architecture, or bacterial cell–cell proximity, thereby favoring persistence or virulence.

A periodontal disease context could magnify these effects. Periodontitis increases plaque mass, pocket depth, inflammatory exudation, proteolysis, and local ecological instability. Saliva and subgingival plaque also exhibit coordinated microbial changes across disease states [21,22]. In that setting, a retained plastic particle could serve as a scaffold, a sorbent surface, and an inflammatory cofactor simultaneously. The subgingival oral biofilm may therefore be especially vulnerable to plastic-mediated ecological amplification.

This framing must be stated carefully. No direct evidence currently demonstrates an oral plastisphere in human subgingival plaque; there are no PubMed-indexed studies showing that plastic particles increase virulence, antibiotic resistance gene (ARG) transfer, or pathogen selection within authentic human oral biofilms. The oral plastisphere concept is therefore presented here as a testable hypothesis grounded in environmental biofilm data and compatible with periodontal biology, not as a demonstrated phenomenon. Direct validation in multi-species subgingival biofilm models and contamination-controlled human sampling (Section 11) is required before this framing can be treated as established.

## 8. Bacterial Degradation of Plastics and the Special Case of Dental Polymers

A central theme is whether bacteria degrade plastics. Scientifically, this question needs to be split into two domains. The first concerns the biodegradation of environmental plastics, such as polyethylene, polystyrene, and other commodity polymers, by environmental or opportunistic microbes. The second concerns the biodegradation of dentistry-related methacrylate polymers by oral bacteria and saliva. The second domain is supported far more directly by oral evidence.

Delaviz et al. reviewed the biodegradation of resin composites and adhesives by oral bacteria and saliva, concluding that restorative material design should account for the degradative conditions of the oral environment [12]. Cariogenic bacteria can degrade dental resin composites and adhesives [13], and *Enterococcus faecalis* possesses esterase-like activity capable of hydrolyzing methacrylate-based dental resins [14]. These studies establish that oral microorganisms do not merely attach to polymeric materials; they can chemically transform them.

This matters for biofilm pathogenicity because the interaction is reciprocal. Degradation products from BisGMA-based composite resin can modulate *Streptococcus mutans* gene expression related to biofilm formation and virulence [31]. The implication is powerful: polymer degradation can reshape microbial behavior, while microbial growth can accelerate it. In the mouth, that feedback loop may increase interfacial failure, microbial colonization, and inflammatory stimulation at restoration margins.

What remains unproven is whether core periodontal consortia can substantially degrade common environmental MNPs in vivo within plaque or periodontal pockets. The current evidence more securely supports a hybrid model: oral microbes are well documented to degrade methacrylate dental polymers, and plastic-degrading capacity in other bacteria can enhance pathogenicity; therefore, reciprocal microbe–polymer interactions in the oral cavity are real, but their extent relative to environmental microplastics in periodontitis remains to be quantified.

## 9. Micro-/Nanoplastics, Chronic Disease, and the Possible Oral-Systemic Bridge

The periodontium is not the only site in which MNPs intersect with chronic disease biology. Human biomonitoring studies have detected microplastics in the placenta, blood, and arterial tissue, indicating that internal exposure and tissue distribution are no longer hypothetical. Microplastics have been identified in human placental specimens using pyrolysis gas chromatography mass spectrometry [32]. Plastic particle pollution in human blood has been reported, including polyethylene terephthalate, polyethylene, and polystyrene [33], and quantitative blood analysis has been further advanced using non-targeted pyrolysis GC-MS [33]. Microplastics have also been detected in human arteries, with higher levels in arteries containing atherosclerotic plaques than in plaque-free aortic tissue [34].

Mechanistic studies support the plausibility of chronic disease amplification once particles access internal tissues. Chen et al. showed that oral exposure to microplastics can injure the colon via oxidative stress, immune imbalance, and barrier dysfunction [27]. Bishop et al. showed that authentic environmental MNPs provoke inflammatory cytokine release and cell death in human cells [26], while Skaba et al. summarized evidence that nanoplastics can cross biological barriers and disrupt immune homeostasis [28]. Bruno et al. reviewed how orally ingested MNPs may contribute to inflammatory bowel disease and colorectal carcinogenesis by inducing chronic epithelial injury, disrupting mucus, and exerting systemic toxic effects [35].

Beyond gut and vascular targets, MNPs are increasingly implicated in metabolic dysregulation with direct relevance to the periodontal context. Laboratory evidence describes how polystyrene MNPs can disrupt glucose metabolism and promote insulin resistance pathways, with worsening effects in the setting of high-fat diet-induced dysbiosis and barrier disruption [36]. While epidemiological proof of a causal link between MNP exposure and type 2 diabetes in humans remains absent, this mechanistic profile is relevant here because periodontitis and type 2 diabetes share a well-characterized bidirectional inflammatory relationship, with overlapping mediators—including IL-6, TNF-α, and RANKL—contributing to both periodontal tissue destruction and insulin resistance [37]. A periodontitis–MNP interaction model could therefore plausibly amplify metabolic inflammatory burden through this oral–metabolic axis, representing a convergence point that longitudinal cohort studies in people with both periodontitis and diabetes risk could directly test.

These observations matter for periodontal science because periodontitis already has a recognized oral–systemic component [37]. Experimental animal work has established mechanistic causality for at least some systemic effects: swallowed *P. gingivalis* can alter gut microbiota composition, increase intestinal permeability, and drive systemic endotoxemia through the oral–gut axis, while periodontal-specific Th17 cells expanded during disease can migrate to the gut and contribute to colitis [37]. This oral–gut–systemic inflammatory transmission route is one that MNP retention at the periodontium could plausibly intensify if particles or polymer degradation products enter the swallowed salivary stream. A chronically inflamed periodontal interface may release cytokines, bacterial products, or whole microbes into the circulation. If that same interface also retains or reacts to MNPs, a combined exposure model becomes conceivable: periodontal disease may increase local particle persistence and inflammatory responsiveness, while particle exposure may intensify tissue injury, thereby amplifying systemic spillover [38].

The oral-systemic bridge proposed here is biologically coherent but remains empirically unvalidated. No longitudinal human studies have tested whether higher oral MNP burdens are associated with accelerated periodontal breakdown, elevated circulating inflammatory markers, or increased incidence of cardiovascular, metabolic, or other chronic outcomes. Cross-sectional detection of MNPs in blood, placenta, and arterial tissue establishes systemic exposure, but does not link that burden to oral sources or to periodontal status. Closing this gap requires prospective cohorts that measure oral polymer burden, periodontal parameters, systemic inflammatory biomarkers, and clinical outcomes over time (Section 11). Until such data exist, the oral-systemic MNP hypothesis should be treated as a research priority rather than an established risk pathway.

## 10. Evidence Synthesis: What Is Established, What Is Plausible, and What Remains Unresolved

Three conclusions can be stated with confidence. First, human oral retention of plastic particles occurs, at least in dental calculus [15]. Second, oral and dental materials can serve as local sources of MNPs or polymer degradation products, and oral microorganisms can degrade methacrylate polymers [11,12,13,14]. Third, MNPs activate inflammatory pathways that overlap extensively with those implicated in periodontitis, including NF-κB, IL-1β, IL-6, oxidative stress, and immune-cell reprogramming [15,16,28].

Three additional conclusions are biologically plausible but not yet definitively proven in humans. Oral biofilms likely retain plastic particles more broadly than calculus alone; plastic particles embedded in oral biofilms may increase pathogenicity, resilience, and inflammatory potential; and periodontitis may increase oral retention and possibly systemic dissemination of MNPs due to dysbiosis, calculus accumulation, and chronic tissue inflammation. Each of these ideas is strongly compatible with the current literature, especially environmental plastisphere studies, but each requires direct oral validation [17,18,19,20].

Two claims should presently be delayed. One is that environmental MNP exposure has already been proven to cause human periodontitis [38]. The other is that plastic-driven changes in the virulence of oral biofilms have already been demonstrated in subgingival communities. Both may ultimately prove true, but current evidence does not justify these conclusions.

An evidence-based appraisal of the proposed connection between periodontal micro-/nanoplastics is summarized in Table 1. The principal points of mechanistic convergence are summarized in Table 2.

**Table 1:** Evidence appraisal for the proposed connection between periodontal micro-/nanoplastics.

Claim	Current Evidence Base	Strength of Support	Interpretation
Human oral retention of MNPs occurs	Direct human evidence	Moderate-to-high	Microplastics detected in human dental calculus; strongest direct oral retention evidence [16].
MNPs can activate periodontal cell inflammatory pathways	Direct in vitro periodontal-cell evidence	Moderate	Polyethylene reduced gingival fibroblast viability, impaired migration, and activated NF-κB/IL-1β/IL-6 signaling [16].
The oral cavity is a relevant exposure and generation site	Direct oral exposure and dental material evidence	Moderate	Chewing gum, oral-care products, orthodontic materials, and dental polymers can release particles or polymer by-products [10,11,15].
Plastic-associated biofilms can become more pathogenic or resilient	Strong non-oral experimental evidence; oral extrapolation	Moderate for general plastisphere biology; low-to-moderate for oral-specific translation	Biofilm promotion, EPS increase, quorum sensing activation, ARG transfer, and pathogen persistence reported in environmental systems [18,19,20,21].
Oral microbes degrade dental polymers, and polymer by-products can reshape biofilms	Direct oral microbiology and dental material evidence	High for methacrylate dental polymers	Cariogenic bacteria and *E. faecalis* degrade resins; BisGMA degradation products alter *S. mutans* virulence-related gene expression [13,14,30].
Environmental MNP exposure causes human periodontitis	No direct longitudinal clinical proof	Insufficient	Currently unproven; supported mainly by mechanistic plausibility and limited direct oral evidence [38].

Strength of support labels follow the three-tier framework defined in Section 2. *High* = direct human or human-cell evidence in an oral or periodontal context with independent replication. *Moderate* = direct evidence from a single primary oral study, or consistent mechanistic evidence from closely adjacent human systems. *Low-to-moderate* = mechanistic evidence from environmental or non-oral biological systems requiring direct oral validation. *Insufficient* = claim depends on longitudinal or interventional human data that do not yet exist.

**Table 2:** Mechanistic convergence between micro-/nanoplastic biology and periodontal pathobiology.

Pathway	MNP-Associated Evidence	Periodontal Relevance	Implication
Oxidative stress and NF-κB activation	Inflammatory signaling and redox imbalance after MNP exposure in oral and non-oral systems	Central to periodontal tissue destruction and host dysregulation	Shared pathway likely to intensify chronic inflammatory signaling
Inflammasome activity and IL-1β/IL-6 release	Gingival fibroblast and human cell studies show cytokine upregulation; calculus itself activates NLRP3	IL-1β and IL-6 are core mediators of periodontitis and oral-systemic inflammation	Particle exposure may amplify an already activated periodontal cytokine network
Impaired migration, apoptosis, and wound repair	Polyethylene reduced fibroblast viability and migration; barrier injury was reported in gut models	Delayed epithelial/connective tissue repair supports lesion chronicity	MNPs may hinder resolution after periodontal injury or therapy
Macrophage and T-cell polarization	Orthodontic particles altered macrophage homeostasis; non-oral models show immune imbalance	Periodontitis is shaped by maladaptive innate and adaptive immunity	Combined exposure may favor persistent proinflammatory cell states
Biofilm EPS production, quorum sensing, and ARG transfer	Plastic particles enhance EPS, virulence traits, and horizontal gene transfer in environmental biofilms	Oral biofilms depend on matrix architecture, signaling, and coaggregation	Potential oral plastisphere mechanism requiring direct subgingival testing
Barrier dysfunction and translocation potential	Systemic tissue studies support internal exposure and barrier injury	Periodontal tissues already form an inflamed, permeable interface	Could increase the dissemination of inflammatory mediators, microbes, or particles

## 11. Priority Research Agenda for Environmental Oral Microbiology

The most urgent need is contamination-controlled oral biomonitoring across periodontal health states. Studies should quantify and polymer-profile MNPs in saliva, supragingival plaque, subgingival plaque, gingival crevicular fluid, dental calculus, and—where ethically feasible—gingival tissue, with rigorous field blanks and source controls. Participant-level information on restorations, aligners, retainers, occupational exposure, diet, smoking, and oral-care products should be recorded to distinguish environmental and dentistry-derived polymers.

Second, the field needs spatially resolved biofilm science. Raman microspectroscopy, pyrolysis GC-MS, high-resolution imaging, and correlative microscopy should be used to determine whether particles are merely present in plaque or whether they are actually embedded within matrix-rich microcolonies, concentrated near inflammatory fronts, or associated with particular taxa. Periodontal pocket models should examine whether particle size, charge, weathering state, and adsorbed salivary proteins alter coaggregation, EPS composition, redox gradients, and antimicrobial tolerance.

Third, multi-species oral biofilm models should replace single-species convenience models whenever possible. Pseudomonas is informative mechanistically, but oral translation requires subgingival consortia that include pathobionts, bridge species, and health-associated taxa. Endpoints should include transcriptional virulence signatures, proteolysis, lipopolysaccharide burden, short-chain fatty acid production, host-cell invasion, and osteoimmune signaling in co-culture with gingival epithelial cells, fibroblasts, neutrophils, and macrophages.

Fourth, longitudinal clinical studies are essential. Cross-sectional detection alone cannot establish whether higher oral MNP burdens precede disease progression or simply accumulate in already diseased mouths. Ideal designs would track polymer burdens, periodontal parameters, inflammatory biomarkers, microbiome states, and systemic markers, such as circulating cytokines, over time, while also documenting major confounders, including periodontal therapy, dietary patterns, and changes in dental materials.

Finally, intervention studies deserve attention. If calculus is an exposure archive and an inflammatory substrate, then mechanical debridement and periodontal treatment may reduce the local MNP burden, or at least its biologically active matrix context. Likewise, source-reduction strategies—such as lower-shedding orthodontic materials, less abrasive polymers, or improved restorative chemistries—may represent a pragmatic translational path even before large-scale causal certainty is achieved.

**Figure 1:**
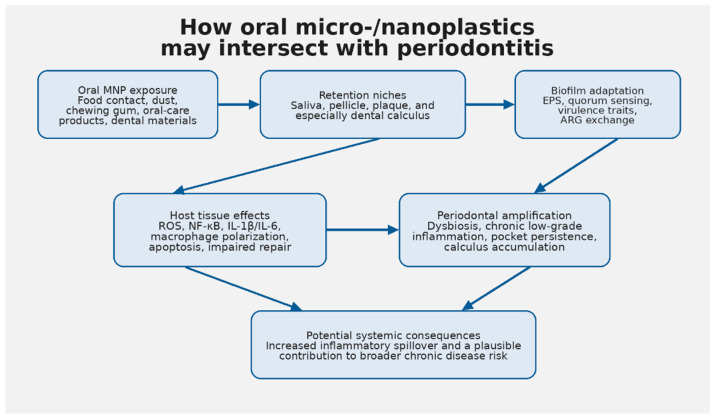
Proposed pathway linking oral micro-/nanoplastic exposure to periodontal amplification and possible systemic effects.

To improve practical applicability, we suggest the following prioritization across the research agenda. The highest near-term priority is contamination-controlled oral biomonitoring stratified by periodontal status, because it is technically feasible with existing IRB-managed clinical sampling and Raman or pyrolysis GC-MS platforms, and its output directly enables every downstream question. Second-tier priorities are multi-species oral biofilm models and polymer source attribution (environmental versus dentistry-derived), both of which are feasible within 1 to 2 years in most translational labs and are essential for mechanistic causality. Third-tier priorities are longitudinal clinical cohorts and intervention studies, which carry the highest translational impact but require multi-year timelines, larger budgets, and established biomonitoring protocols as prerequisites. Spatially resolved biofilm imaging spans tiers: feasible in well-equipped labs immediately, but highest impact when paired with biomonitoring output. This sequencing is reflected in Table 3. The overall exposure-retention-amplification model synthesized in this review is summarized in Figure 1.

## 12. Conclusions

The emerging literature supports a coherent yet incomplete model linking periodontitis, chronic low-grade inflammation, and MNP exposure. The mouth is not merely a passive transit site for plastic particles; it is an ecologically complex and disease-sensitive environment that can retain particles, generate plastic debris from dental materials, and convert particle exposure into host inflammatory signaling. The discovery of diverse microplastics in human dental calculus and the demonstration that polyethylene can injure gingival fibroblasts provide the clearest direct oral evidence to date [15].

Periodontitis adds an important layer of biological plausibility by amplifying biofilm mass, inflammatory signaling, and tissue vulnerability. The environmental biofilm literature further suggests that plastic particles can serve as scaffolds for pathogenic adaptation, ARG exchange, and resilience, raising the possibility of an oral plastisphere within plaque and calculus. Oral bacteria have already been shown to degrade methacrylate dental polymers, indicating that reciprocal interactions between plastic substrates and the oral microbiota are a present clinical reality, not a theoretical abstraction.

Even so, the literature does not yet justify the claim that environmental MNP exposure causes human periodontal disease. The most defensible conclusion is narrower and stronger: current evidence supports a biologically plausible model in which periodontitis may increase local retention, cellular exposure, and perhaps systemic consequences of MNPs while plastic particles and polymer degradation products may worsen inflammatory signaling, biofilm pathogenicity, and tissue-repair failure. That model is now mature enough to deserve direct testing in environmental oral microbiology.

Conceptual summary: exposure sources may feed oral retention niches; retained particles may alter biofilm ecology and host inflammatory signaling; in susceptible individuals, those changes may reinforce chronic periodontitis and broader inflammatory risk.

## Figures and Tables

**Table 3:** Priority research agenda for environmental oral microbiology.

Research Question	Recommended Design	Key Measurements	Why It Matters	Priority Tier
How common are MNPs in periodontal compartments?	Cross-sectional, contamination-controlled clinical sampling	Saliva, supra-/subgingival plaque, GCF, calculus, tissue; polymer fingerprinting; dental-material inventory	Establishes prevalence and source attribution by periodontal status	1 (near-term foundational)
Where are particles located within oral biofilms?	Spatial imaging and correlative spectroscopy	Raman/FTIR/Py-GC-MS plus microscopy; matrix localization; particle size and charge	Determines whether particles are embedded in pathogenic biofilm microdomains	2 (mid-term, cross-tier)
Do MNPs worsen dysbiosis or host injury?	Multi-species oral biofilm and host co-culture models	EPS, quorum sensing, virulence genes, invasion, cytokines, osteoimmune readouts	Tests causality in orally relevant systems rather than environmental surrogates	2 (mid-term)
Are external and dentistry-derived plastics biologically distinct?	Comparative polymer-source studies	Environmental weathered particles versus orthodontic, restorative, and oral-care derived particles	Separates environmental exposure from treatment-related polymer burdens	2 (mid-term)
Does oral plastic burden predict periodontal progression?	Prospective longitudinal cohorts	Periodontal parameters, plastic burden, inflammatory biomarkers, restorations, diet, smoking, therapy history	Moves the field from plausibility to temporality and risk estimation	3 (long-term, high-impact)
Can intervention reduce plastic-associated inflammatory burden?	Clinical or translational intervention studies	Debridement, source reduction, lower-shedding materials, post-treatment polymer measurements	Provides immediate preventive and materials-science relevance	3 (long-term, translational

## Data Availability

No new data were created or analyzed in this narrative review.

## Abbreviations

The following abbreviations are used in this manuscript: ARG, antibiotic resistance gene; EPS, extracellular polymeric substance; GCF, gingival crevicular fluid; MNP(s), micro-/nanoplastic(s); NF-κB, nuclear factor kappa B; ROS, reactive oxygen species.

## References

[1] Di Spirito F., Folliero V., Di Palo M.P., De Benedetto G., Aulisio L., Martina S., Rinaldi L., Franci G. (2025). Micro- and Nanoplastics and the Oral Cavity: Implications for Oral and Systemic Health, Dental Practice, and the Environment—A Narrative Review. J. Funct. Biomater..

[2] Zarus G.M., Muianga C., Hunter C.M., Pappas R.S. (2021). A Review of Data for Quantifying Human Exposures to Micro and Nanoplastics and Potential Health Risks. Sci. Total Environ..

[3] Pant U., Tate J., Liu X., Birse N., Elliott C., Cao C. (2025). From Automated Raman to Cost-Effective Nanoparticle-on-Film (NPoF) SERS Spectroscopy: A Combined Approach for Assessing Micro- and Nanoplastics Released into the Oral Cavity from Chewing Gum. J. Hazard. Mater..

[4] Leslie H.A., van Velzen M.J.M., Brandsma S.H., Vethaak A.D., Garcia-Vallejo J.J., Lamoree M.H. (2022). Discovery and Quantification of Plastic Particle Pollution in Human Blood. Environ. Int..

[5] Cox K.D., Covernton G.A., Davies H.L., Dower J.F., Juanes F., Dudas S.E. (2019). Human Consumption of Microplastics. Environ. Sci. Technol..

[6] Senathirajah K., Attwood S., Bhagwat G., Carbery M., Wilson S., Palanisami T. (2021). Estimation of the Mass of Microplastics Ingested—A Pivotal First Step towards Human Health Risk Assessment. J. Hazard. Mater..

[7] Cecoro G., Annunziata M., Iuorio M.T., Nastri L., Guida L. (2020). Periodontitis, Low-Grade Inflammation and Systemic Health: A Scoping Review. Medicina.

[8] Mazurek-Mochol M., Bonsmann T., Mochol M., Poniewierska-Baran A., Pawlik A. (2024). The Role of Interleukin 6 in Periodontitis and Its Complications. Int. J. Mol. Sci..

[9] Torrungruang K., Vathesatogkit P., Mahanonda R., Thienpramuk L. (2024). Periodontitis and Hypertension Are Linked through Systemic Inflammation: A 5-Year Longitudinal Study. J. Clin. Periodontol..

[10] Saha U., Jena S., Simnani F.Z., Singh D., Choudhury A., Naser S.S., Lenka S.S., Kirti A., Nandi A., Sinha A. (2025). The Unseen Perils of Oral-Care Products Generated Micro/Nanoplastics on Human Health. Ecotoxicol. Environ. Saf..

[11] Delaviz Y., Finer Y., Santerre J.P. (2014). Biodegradation of Resin Composites and Adhesives by Oral Bacteria and Saliva: A Rationale for New Material Designs That Consider the Clinical Environment and Treatment Challenges. Dent. Mater..

[12] Bourbia M., Ma D., Cvitkovitch D.G., Santerre J.P., Finer Y. (2013). Cariogenic Bacteria Degrade Dental Resin Composites and Adhesives. J. Dent. Res..

[13] Marashdeh M.Q., Gitalis R., Levesque C., Finer Y. (2018). *Enterococcus faecalis* Hydrolyzes Dental Resin Composites and Adhesives. J. Endod..

[14] Warunek J., Warunek S., Calderon M., Franks J., Watkins S., Turnquist H., Al-Jewair T. (2026). Orthodontic Derived Microplastics Impact Macrophage Differentiation and Homeostasis. Prog. Orthod..

[15] Wu Z., Yi Y., Yang B., Cui X., Chen F., Wu G. (2025). Polyethylene: An Identified Component of Human Dental Calculus Triggers Cytotoxicity and Inflammatory Responses in Gingival Fibroblasts. Environ. Int..

[16] Mahmud F., Sarker D.B., Jocelyn J.A., Sang Q.-X.A. (2024). Molecular and Cellular Effects of Microplastics and Nanoplastics: Focus on Inflammation and Senescence. Cells.

[17] Huang P., Li Z., Liu R., Bartlam M., Wang Y. (2024). Polystyrene Nanoparticles Induce Biofilm Formation in *Pseudomonas aeruginosa*. J. Hazard. Mater..

[18] Zhou Y., Zhang G., Zhang D., Zhu N., Bo J., Meng X., Chen Y., Qin Y., Liu H., Li W. (2024). Microplastic Biofilms Promote the Horizontal Transfer of Antibiotic Resistance Genes in Estuarine Environments. Mar. Environ. Res..

[19] Wang H., Chen H., Ruan C., Liao J., Schwarz C., Shi B., Alvarez P.J.J., Yu P. (2026). Nanoplastics Induce Prophage Activation and Quorum Sensing to Enhance Biofilm Mechanical and Chemical Resilience. Water Res..

[20] Zhang X., Dong Z., Zhang S., Ma J., Liu S. (2026). Microplastic Biofilm as Hotspots of Antibiotic Resistance Genes and Potential Pathogens. npj Biofilms Microbiomes.

[21] Belstrøm D., Constancias F., Drautz-Moses D.I., Schuster S.C., Veleba M., Mahé F., Givskov M. (2021). Periodontitis Associates with Species-Specific Gene Expression of the Oral Microbiota. npj Biofilms Microbiomes.

[22] Jung J.-S., Kook J.-K., Park S.-N., Lim Y.K., Choi G.H., Kim S., Ji S. (2024). Salivary Microbiota Reflecting Changes in Subgingival Microbiota. Microbiol. Spectr..

[23] Hajishengallis G. (2015). Periodontitis: From Microbial Immune Subversion to Systemic Inflammation. Nat. Rev. Immunol..

[24] Montenegro Raudales J.L., Yoshimura A., Sm Z., Kaneko T., Ozaki Y., Ukai T., Miyazaki T., Latz E., Hara Y. (2016). Dental Calculus Stimulates Interleukin-1β Secretion by Activating NLRP3 Inflammasome in Human and Mouse Phagocytes. PLoS ONE.

[25] Velsko I.M., Overmyer K.A., Speller C., Klaus L., Collins M.J., Loe L., Frantz L.A.F., Sankaranarayanan K., Lewis C.M., Rodriguez Martinez J.B. (2017). The Dental Calculus Metabolome in Modern and Historic Samples. Metabolomics.

[26] Bishop B., Webber W.S., Atif S.M., Ley A., Pankratz K.A., Kostelecky R., Colgan S.P., Dinarello C.A., Zhang W., Li S. (2025). Micro- and Nano-Plastics Induce Inflammation and Cell Death in Human Cells. Front. Immunol..

[27] Chen J., Cheng Y., Fu R., Chen X., Zhang P., Lu Y., Liu B., Chen P., Wang J., Cao H. (2025). Multiomics Reveals Nonphagocytosable Microplastics Induce Colon Inflammatory Injury via Bile Acid–Gut Microbiota Interactions and Barrier Dysfunction. ACS Appl. Mater. Interfaces.

[28] Skaba D., Fiegler-Rudol J., Dembicka-Mączka D., Wiench R. (2025). Nanoplastics and Immune Disruption: A Systematic Review of Exposure Routes, Mechanisms, and Health Implications. Int. J. Mol. Sci..

[29] Ormsby M.J., Woodford L., White H.L., Fellows R., Quilliam R.S. (2024). The Plastisphere Can Protect *Salmonella* Typhimurium from UV Stress under Simulated Environmental Conditions. Environ. Pollut..

[30] Howard S.A., de Dios R., Maslova E., Myridakis A., Miller T.H., McCarthy R.R. (2025). *Pseudomonas aeruginosa* Clinical Isolates Can Encode Plastic-Degrading Enzymes That Allow Survival on Plastic and Augment Biofilm Formation. Cell Rep..

[31] Singh J., Khalichi P., Cvitkovitch D.G., Santerre J.P. (2009). Composite Resin Degradation Products from BisGMA Monomer Modulate the Expression of Genes Associated with Biofilm Formation and Other Virulence Factors in *Streptococcus mutans*. J. Biomed. Mater. Res. A.

[32] Garcia M.A., Liu R., Nihart A., El Hayek E., Castillo E., Barrozo E.R., Suter M.A., Bleske B., Scott J., Forsythe K. (2024). Quantitation and Identification of Microplastics Accumulation in Human Placental Specimens Using Pyrolysis Gas Chromatography Mass Spectrometry. Toxicol. Sci..

[33] Nijenhuis W., Houthuijs K.J., Brits M., van Velzen M.J.M., Brandsma S.H., Lamoree M.H., Béen F.M. (2025). Improved Multivariate Quantification of Plastic Particles in Human Blood Using Non-Targeted Pyrolysis GC-MS. J. Hazard. Mater..

[34] Liu S., Wang C., Yang Y., Du Z., Li L., Zhang M., Ni S., Yue Z., Yang K., Wang Y. (2024). Microplastics in Three Types of Human Arteries Detected by Pyrolysis-Gas Chromatography/Mass Spectrometry (Py-GC/MS). J. Hazard. Mater..

[35] Hajishengallis G., Chavakis T. (2021). Local and Systemic Mechanisms Linking Periodontal Disease and Inflammatory Comorbidities. Nat. Rev. Immunol..

[36] Hsiao H.-Y., Nien C.-Y., Shiu R.-F., Chin W.-C., Yen T.-H. (2025). Microplastic and Nanoplastic Exposure and Risk of Diabetes Mellitus. World J. Clin. Cases.

[37] Bruno A., Dovizio M., Milillo C., Aruffo E., Pesce M., Gatta M., Chiacchiaretta P., Di Carlo P., Ballerini P. (2024). Orally Ingested Micro- and Nano-Plastics: A Hidden Driver of Inflammatory Bowel Disease and Colorectal Cancer. Cancers.

[38] Francis D.L., Reddy S.S.P. (2025). Microplastics in the Pathogenesis of Periodontal Diseases: A Narrative Review. Ann. Glob. Health.

